# The application of endoscopic debridement combined with metagenomic next-generation sequencing technology in primary spinal infections: a retrospective study

**DOI:** 10.1186/s13018-024-05385-5

**Published:** 2025-02-25

**Authors:** Xiaofei Feng, Jie Cheng, Luyong Jiang, Jiayi Lin, Zhewei Ye, Qingjiang Pang, Jiangtao Liu

**Affiliations:** 1 Department of Orthopaedics, Wuhan Union Hospital, Tongji Medical College, Huazhong University of Science and Technology, Wuhan, 430022 China; 2 Department of Orthopedics, Ningbo No. 2 Hospital, Ningbo, 315000 China; 3 Cixi Biomedical Research Institute, Wenzhou Medical University, Zhejiang, 315000 China

**Keywords:** Spinal infection, Spinal endoscopy, Metagenomic next-generation sequencing, Etiological diagnosis

## Abstract

**Purpose:**

Spinal endoscopy is a novel minimally invasive spinal surgery technique used in recent years to treat various degenerative spinal diseases. Metagenomic next-generation sequencing (mNGS) is a new method for identifying infectious microorganisms in infectious diseases. We aim to evaluate the application effect of combining spinal endoscopy with mNGS in diagnosing and treating spinal infections.

**Methods:**

The clinical data of 62 patients with suspected spinal infectious diseases admitted from January 2020 to December 2023 were retrospectively analyzed. All patients underwent spinal endoscopy to obtain tissue specimens, histopathological examination, routine bacterial culture, and mNGS sequencing. Describe the pathogenic microbial spectrum of spinal infection, and compare the differences in sensitivity (true positive rate) and specificity (true negative rate) between the two detection methods. White blood cell (WBC) erythrocyte deposition rate (ESR), C-reactive protein (CRP), visual analog scale (VAS), Japanese Orthopaedic Association (JOA) score, Oswestry Disability Index (ODI), and other clinical results were analyzed.

**Results:**

In 62 cases, mNGS, microbiological culture, serologic testing, and pathologic examination results were obtained. 49 cases of spinal infections and 13 cases of non-spinal infections were finally diagnosed clinically. Among the 49 patients with spinal infections, there were 31 cases of purulent bacterial infections, 8 cases of tuberculosis infections, and 10 cases of infections with unspecified etiological microorganisms. Among the 13 cases of non-spinal infections, there were 3 cases of spinal tumors, 6 cases of Modic changes of the endplates, and 4 cases of endplate fracture. The positive rate of microbial culture was 36.73% (18/49), and the positive rate of the mNGS test was 71.43% (35/49), which was statistically different from each other (*P* < 0.01). The sensitivity of the mNGS test was 71.43%, and the specificity of the mNGS test was 84.62%. At the 3-month follow-up, WBC, ESR, and CRP levels were normalized. The VAS, JOA score, and ODI of the lower back and legs at each follow-up point after surgery were significantly improved compared with those before surgery, and the difference was statistically significant (*P* < 0.01).

**Conclusion:**

Metagenomic sequencing technology is fast, efficient, and accurate in detecting pathogenic microorganisms, and has high diagnostic value in the diagnosis and treatment of spinal infections. Spinal endoscopic debridement combined with mNGS can achieve good clinical results.

## Introduction

Spinal infectious diseases are caused by pathogenic microorganisms that settle in the spinal column area and spread from the affected segment to adjacent segments or tissues [1]. It often affects the vertebral body, intervertebral disc, or paravertebral tissue, including vertebral osteomyelitis, discitis, and epidural abscess. According to the types of pathogenic microorganisms, infections are classified as specific and nonspecific infections. Specific infections mainly refer to infections such as tuberculosis or non-tuberculous mycobacteria, fungi, Brucella, etc.; nonspecific infections mainly refer to purulent infections caused by G^−^ or G^+^ bacteria, such as Staphylococcus aureus, Escherichia coli, and various streptococci [2–4].

Clinical manifestations, laboratory tests, imaging findings, and histopathological examinations of spinal infections caused by different pathogens lack specificity. Identifying the pathogenic microorganism is crucial for diagnosing the infection [5]. Therefore, seeking a simpler and more efficient method to obtain the pathogenic microorganism for early diagnosis is necessary. However, traditional detection methods mainly include culture and molecular biology tests. Although a positive culture is considered the gold standard for diagnosis, this method has shortcomings such as long culture periods, low positivity rates, and high false positive rates. Literature reports a blood culture positivity rate of 30–50% for spinal infectious diseases [6–8]. While the positive rate of culture from biopsy specimens of lesions is less than 60% [9, 10]. PCR, as a representative of molecular biology detection, has high sensitivity and specificity but requires the pre-designing of primers, only targeting specific pathogens, which is of great clinical significance for the diagnosis of spinal tuberculosis-related infections. Literature reports that traditional methods rely on diagnosing spinal infectious diseases still cannot definitively identify 25%~60% of the infectious pathogens [11–15]. Therefore, there is an urgent need in clinical practice for a broad-spectrum screening detection method for non-tuberculous infectious pathogens.

Metagenomic next-generation sequencing technology, as an emerging pathogen detection method, is a new sequencing method relative to first-generation DNA sequencing technology. It can detect various pathogens without bias, making up for the shortcomings of various traditional pathogen detection methods. This technology features fast speed, high accuracy, high positivity rate, and broad coverage of pathogenic bacteria. It has unique advantages in detecting rare pathogens such as mycoplasma, fungi, and brucella. Currently, it has been widely used in the detection of blood, respiratory tract, cerebrospinal fluid, and other specimens, achieving certain effectiveness [15]. However, there are relatively few clinical reports on the diagnosis of infectious diseases in bone and joints, mainly based on single-center, small-sample research reports.

The traditional treatment modalities for spinal infections include mainly non-surgical and open-surgical treatments. Some scholars believe that most intervertebral space infections can be cured by non-surgical treatment through absolute bed rest, strengthening nutrition, and symptomatic treatment with large doses of sensitive antibacterial drugs [16]. However, non-surgical treatment can lead to long bed rest, easy to prolong the course of the disease, and easy to lead to lumbar instability and pseudoarthrosis in the later stage [17]. With the advancement of spinal surgery technology and the development of minimally invasive concepts, intervertebral endoscopic has been widely used in the treatment of lumbar spine diseases, and it has a greater advantage in the treatment of spinal infections.

This study analyzed the use of metagenomic next-generation sequencing (mNGS) for diagnosing spinal infectious diseases. The goal was to assess mNGS’s effectiveness, advantages, and limitations in diagnosing spinal infections. Additionally, the study aimed to identify factors that affect the detection of pathogens in samples to improve the accuracy of results, reduce false positives and false negatives, and evaluate the potential benefits of combining mNGS with endoscopic treatment for primary spinal infections.

## Materials and methods

### Patients

This study is a retrospective research based on the data of inpatients in our hospital. It involves collecting data on all patients suspected of spinal infection who underwent spinal endoscopy sampling and mNGS diagnosis from January 2020 to December 2023. Incorporation criteria: (1) Suspected spinal infectious diseases diagnosed based on clinical manifestations, imaging, and laboratory examinations, (2)Obtained lesion specimens under spinal endoscopy, (3)Perform mNGS testing and routine laboratory inspection. Exclusion criteria: (1) Not completing all routine tests and mNGS, (2) Specimens were significantly contaminated during sampling, transportation, and handling, or the cold chain transportation failed, (3) Incomplete or unclear medical history. According to the above inclusion and exclusion criteria, a total of 62 patients were included in this study. This study was approved by the Ethics Committee of Ningbo No.2 Hospital, and all patients signed informed consent.

### Blood tests

**Fig. 1:**
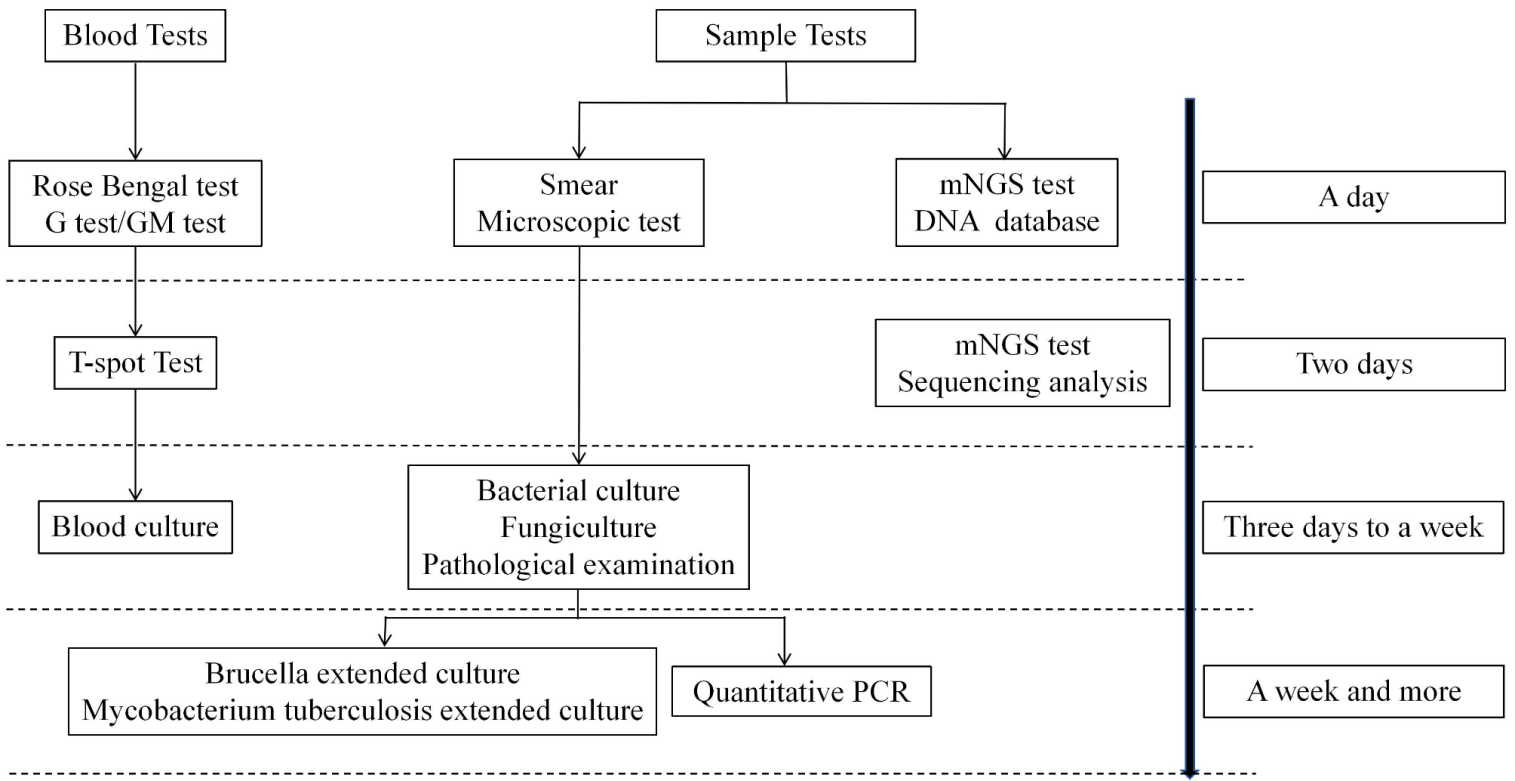
Laboratory examination and aging chart of patients with spinal infection

Results of the Rose Bengal test, G test, GM test, erythrocyte sedimentation rate (ESR), C-reactive protein (CRP) level, white blood cell (WBC) count, and tuberculosis tests (T-SPOT.TB) were obtained. All blood tests were performed in the laboratory of our hospital. The testing process is shown in Fig. 1.

### Sample collection and tests

All patients obtain blood and tissue specimens from the lesion site. Blood specimens are collected at the hospital, and tests including blood routine, C-reactive protein, erythrocyte sedimentation rate, enzyme-linked immune-spot assay (T-SPOT.TB), detection of glucan content (GM test), and platelet agglutination test are performed for infectious disease laboratory examination. Tissue specimens are obtained directly at the center of the lesion under spinal endoscopy. Fresh samples from all patients are promptly stored in sterile containers and transported to the laboratory on dry ice for metagenomic next-generation sequencing (mNGS).

### Collection of specimens

**Fig. 2:**
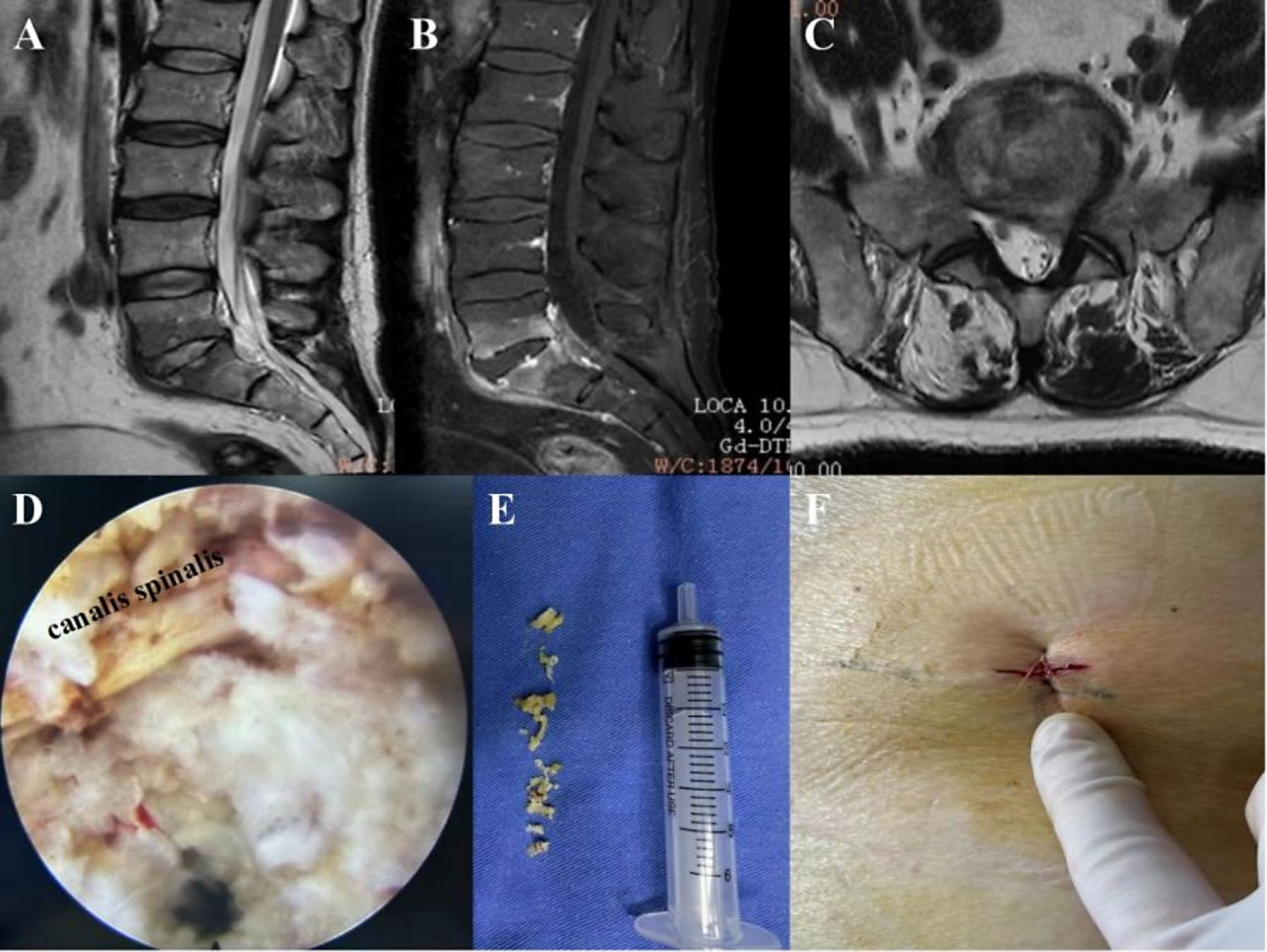
The focal specimens were collected under a spinal endoscope

The spinal endoscopic was advanced into the intervertebral space to monitor lesion clearance and end-plate preparation. The nucleus pulposus was completely removed, the pus in and around the vertebral canal was cleaned, and the damaged and hardened bone was cured. The tissues taken during the operation were stored aseptically and submitted for examination(Figure 2).

### mNGS

The whole process of mNGS testing strictly follows the principle of aseptic operation. The nucleic acid extraction process is carried out in the biosafety cabinet. The operators use the aseptic consumables, reagents, and the gun head with filter elements according to the SOP process.

#### Nucleic acid extraction

Sample 200 µl of nucleic acid was obtained after pretreatment and nucleic acid extraction, refer to the manual (Nucleic acid extraction kit, Hangzhou Jieyi Biotechnology Co. LTD.)

#### Library preparation

(a) Configuration premix solution: fragmentation enzyme 5 µl add fragmentation buffer 10 µl preparation of premix solution, vortexing for 5s to mix the solution, instantaneous centrifugation for 5s and then placed on ice. (b)After opening the cartridge, add 40 µl of purified magnetic beads to test tube No. 1. 1040 µl of absolute ethanol to test tube No. 8. 5 µl of ligase to test tube No. 10. 30 µl of connector to test tube No. 11. 15 µl of premix to test tube No. 12, and 35 µl of nucleic acid extract to test tube No. 3 to avoid bubbles. The library is obtained after setting up the software running program. (c)Library quantification: Library quantification was quantified by quantitative real-time PCR (KAPA) technology. (d)Computer sequencing: High-throughput sequencing on Illumina Nextseq with 75 bp single-end sequencing with about 20 million sequences generated for each library. (e) Bioinformatics analysis: Bioinformatics analysis performed human genome sequence reference (GRCh38.p13) filtering as described in the literature, with reads of the remaining sequences and reference database (NCBI; ftp://ftp.ncbi.nlm.nih.gov/genomes/) to determine species, read count and read count relative abundance. Negative controls were tested in each sequencing, and negative controls used manually cultured 1104 / ml Jurkat tumor cells, excluding environmental bacteria and reagent background bacteria sequences, such as Acinetobacter, Acinetobacter johnsonii, etc.

### Evaluation index

The differences between mNGS and conventional bacterial culture on the sensitivity (true positive rate), specificity (true negative rate), false negative rate, and false negative rate. ESR, CRP, WBC, low back, and leg Visual model scores were analyzed at different time points before and after surgery. VAS, Japanese Orthopaedic Association (JOA) score, Oswestry disability index (Oswestry disability index) ODI) at six-month follow-up.

### Statistical analysis

Statistical analyses were performed using the SPSS 25.0 (SPSS Corporation, USA) statistical software package. An Independent sample t-test was used for comparison between conventional bacterial culture and mNGS. The count data (specimen acquisition method, antibiotics applied within 4 weeks, and specimen type) were expressed by example (%), and the comparison of positive rate was performed by χ2 test. The comparison of the true positive rate, true negative rate, false positive rate, and false negative rate of different detection methods was performed using the χ2 test. ANOVA with one-way repeated measures data was used to compare VAS, JOA scores, ODI, ESR, and CRP at all postoperative time points. The effect of different factors on the detection of pathogenic microorganisms was analyzed by non-conditional logistic regression. A difference of *P* < 0.05 indicated statistical significance.

## Results

### Patient features

Among the 62 patients, 37 were males and 25 were females. Age 14–81 years old (49.4 ± 16.2 y). All pathology specimens were obtained by spinal endoscopic surgery. Combined with the history, clinical manifestations, physical examination, laboratory examination, imaging examination, and intraoperative findings. 49 cases of mNGS were clinically diagnosed as spinal infections and 13 cases were non-spinal infections. Of the 49 cases of spinal infection, 32 were treated with antibiotics before obtaining lesion specimens and 17 were treated without antibiotics. ESR (*P* < 0.001), CRP (*P* < 0.001), and WBC count (*P* < 0.001) are all significantly higher in the infected group (Table 1). There were 4 cases of cervical, 18 cases of thoracic, and 31 cases of lumbar infections. There was no statistical difference in age or gender between the infected and aseptic groups. (Table 2)

**Table 1:** Demographic characteristics of cases included in the study

Characteristic	Aseptic	Infected	*P* value
Age(y)	52.9 ± 11.6	47.1 ± 13.4	0.58
Gender(M/F)	8/5	29/20	13.69
ESR(mm/h)	23.64 ± 14.27	82.65 ± 19.81	<0.01
CRP(mg/L)	9.24 ± 3.71	56.72 ± 27.92	<0.01
WBC(10^9^/L)	4.36 ± 1.57	8.27 ± 3.19	<0.01

**Table 2:** Distribution of results from 62 cases of spinal infection

Clinical symptom	Focal site
Focal pain	cervical vertebra(4) lumbar vertebra(8)
Focal pain and fever	thoracic vertebra(9) lumbar vertebra(17)
Focal pain and lower limb pain	lumbar vertebra(11)

### Diagnostic results of pathogenic microorganisms

#### Pathology and culture results of pathogenic microorganism

Among the 49 cases of spinal infection, there were 31 cases of suppurative bacterial infection, 8 cases of tuberculosis infection, and 10 cases of spinal infection without clear pathogenic microorganisms. There were 13 cases of non-spinal infection, including spinal tumor in 3 cases, endplate Modic change in 6 cases, and endplate fracture in 4 cases. Among the 49 patients with clear evidence of pathogenic microorganisms, 37 cases were identical with the laboratory results and 12 cases were different from the final diagnosis. The consistency between mNGS and laboratory results was 71.43% (35/49). In the test results of mNGS, the common pathogenic microorganisms of suppurative spondylitis were Staphylococcus aureus, Escherichia coli, streptococcus, and fungi. The other common infection type was spinal tuberculosis. 7 cases of tuberculosis infection were detected by pathology and quantitative PCR, and the smear and culture were negative (Table 3).

**Table 3:** Comparison of clinically confirmed pathogen microorganism results with metagenomic sequencing results

Patient ID	Pathogenic microorganisms	Diagnostic Basis	Results of mNGS and sequence number	Antibiotic
1–11	Stapyhlococcus aureus	Bacterial culture	Stapyhlococcus aureus	Penicillin
12–14	MRSA	Bacterial culture	Stapyhlococcus aureus	Vancomycin
15–16	Streptococcus sanguis	Bacterial culture, PCR	Prohyromonas endodontalis, Treponema deticola, Streptococcus sanguis	Penicillin Metronidazole
17	Streptococcus intermedium	Bacterial culture	Streptococcus intermedium	Linezolid, Minocycline
18–19	Streptococcus constellations	Bacterial culture	Streptococcus constellations	Linezolid, Minocycline
20–34	Escherichia coil	Bacterial culture	Escherichia coil	Cephalosporin, Levofloxacin
25–27	Pseidomonas aeruginosa	Blood culture	Pseidomonas aeruginosa	Cephalosporin, Levofloxacin
28–29	Bacteroides fragilis	Blood culture	Bacteroides fragilis	Carbapenems, Metronidazole
30	Aspergillus flavus	Bacterial culture	Aspergillus flavus	Metronidazole
31–39	Mycobacterium tuberculosis	Pathology, PCR	Mycobacterium Tuberculosis complex	Rifampicin, Isoniazid, Ethambutol, Pyrazinamide
40	Unknown pathogen	Pathology	Streptococcus pseudopneumoniae	Rifampicin, Isoniazid, Ethambutol, Pyrazinamide
41	Unknown pathogen	Pathology	Escherichia coli	Vancomycin Carbapenems
42	Unknown pathogen	Pathology	Streptococcus sinensis	Cephalosporin, Levofloxacin
43	Unknown pathogen	Pathology	Staphylococcus epidermidis	Linezolid, Levofloxacin
44–46	Unknown pathogen	Pathology	Staphylococcus aureus	Linezolid, Levofloxacin
47	Unknown pathogen	Pathology	Negative	Vancomycin
48	Unknown pathogen	Pathology	Negative	Cephalosporin, Levofloxacin
49	Unknown pathogen	Pathology	Negative	Carbapenems
50–52	Spinal metastasis	Pathology	Negative	N/A
53–58	Modic changes	Pathology	Negative	N/A
59–62	Endplate fracture	Pathology	Negative	N/A

#### mNGS test results

The differences in sensitivity (true positive rate), specificity (true negative rate), false positive rate, and false negative rate of pathogenic microorganisms detection between mNGS and conventional bacterial culture were compared. The study suggests that the differences in sensitivity and false-negative rate between mNGS and conventional bacterial culture are statistically significant, indicating that mNGS can effectively improve the detection rate of pathogenic microorganisms (Table 4).

**Table 4:** Specificity and sensitivity of mNGS compared with culture

Test result	bacterial culture	mNGS	χ^2^	*P*
sensitivity	36.73%	71.43%	12.73	< 0.001
false negative rate	63.27%	28.57%		< 0.001
false positive rate	46.15%	15.38%		< 0.001
specificity	53.85%	84.62%	11.64	< 0.001

### Comparison of evaluation index at 1 year of follow-up

The patients had immediate relief of lower back pain after surgery. All patients were followed up for 12 to 18 months, with a mean of 14 months. ESR, CRP, and WBC were all reduced to the normal range at 1 month postoperatively. The difference between the preoperative VAS, JOA score, and ODI index on the first day, first month, third month, sixth month, and the last follow-up was statistically significant (*P* < 0.05)(Table 5).

**Table 5:** The VAS scores of low back and low limb pain, JOA score, ODI, ESR, CRP, and WBC were compared between preoperative and postoperative

Test point	VAS (Low back pain)	VAS (Low limb pain)	JOA (score)	ODI (%)	ESR (m/h)	CRP (mg/L)	WBC (10^9^/L)
preoperative	6.34 ± 1.72	4.07 ± 1.14	12.82 ± 4.68	61.36 ± 9.32	97.52 ± 25.48	65.72 ± 29.15	9.67 ± 3.58
Postoperative Day	3.32 ± 0.63	1.87 ± 1.09	-	-	89.15 ± 19.76	63.18 ± 24.76	9.55 ± 4.24
One month after the operative	1.89 ± 0.43	1.32 ± 0.76	21.57 ± 2.89	36.26 ± 8.93	20.17 ± 10.48	8.46 ± 4.27	3.83 ± 1.62
Three months after the operative	1.37 ± 1.65	1.06 ± 0.53	25.28 ± 2.47	27.48 ± 5.95	19.71 ± 8.84	7.98 ± 3.47	3.86 ± 1.06
Six months after the operative	0.79 ± 0.58	0.71 ± 0.47	26.27 ± 3.73	9.85 ± 4.63	21.08 ± 10.67	8.68 ± 4.28	4.02 ± 1.34
One year after the operative	0.25 ± 0.37	0.28 ± 0.24	28.83 ± 4.36	7.92 ± 3.45	18.63 ± 9.76	9.27 ± 3.89	3.87 ± 1.06
*P*	< 0.001	< 0.001	< 0.001	< 0.001	< 0.001	< 0.001	< 0.001

## Discussion

The diagnosis of spinal infections can be challenging. Initially, spinal infections share clinical and imaging similarities with spinal tumors, seronegative spondylitis fractures, and endplate injuries in the early stages, making them difficult to distinguish. Secondly, spinal infections are mainly caused by pyogenic bacteria, Mycobacterium brucei, Mycobacterium tuberculosis, and fungi. The clinical features, signs, and imaging manifestations often lack specificity, making them easily confused. The accurate and efficient diagnosis of spinal infections and other infectious diseases has always relied on high-quality diagnostic techniques. While microbiologic culture is considered the gold standard for diagnosing spinal infections, the positive rate of bacterial culture for Mycobacterium brucei, Mycobacterium tuberculosis, fungi, and anaerobes is extremely low. Serologic testing is a specific test for one or more microorganisms with unique properties. While highly targeted, it is subject to cross-reactivity, changes in the course of the disease, and the influence of background potency, which may result in false positives or false negatives.

Xu reported an overall positive rate of 90.72% for mNGS in 108 cases of spinal infections, which was significantly higher than the 40% for routine cultures [18]. Wang found that the sensitivity of mNGS in the detection of spinal infection pathogens using one- and two-generation high-throughput sequencing techniques was 73.33% [19]. Ma reported that the sensitivity of mNGS in 31 patients with spinal infections was 70.3% and the specificity was 75.0% [20]. In the present study, the sensitivity and specificity of the mNGS assay reached 71.43% and 84.62%, respectively. Similar to the results of other studies, mNGS was significantly superior to a combination of culture, serologic testing, pathology, and PCR assays.

We assumed that the differences in the diagnostic ability of mNGS in different studies are mainly due to the differences in sequencing platforms, testing centers, and sample tissues. mNGS is not culture-dependent, and its detection results only depend on the DNA content of the pathogenic microorganisms at the time of DNA library establishment. For microorganisms that are difficult to isolate DNA from, such as Mycobacterium tuberculosis, Mycobacterium brucei, and other intracellular bacteria or fungi, the optimization of the extraction process and interpretation rules can be used to improve the detection rate. In addition, even if the pathogenic microorganisms die after anti-infection treatment, their DNA remains active for a short period. However, their DNA can retain activity in the short term, and results can still be obtained by mNGS detection. In this study, some patients were still found to have pathogenic microorganisms by mNGS after anti-infective treatment, indicating that mNGS is less affected by anti-infective treatment in the short term than other detection methods. DNA extraction, library construction, sequencing, and analysis of pathogenic microorganisms in all patients in this study were all completed within 48 h. The process was fully completed within 48 h, which is comparable to serological testing and shorter than conventional culture time (3, 7d, and some cultures even need to be extended to 14d). mNGS’s high efficiency and rapidity imply early intervention of the disease and thus reduce the risk of exacerbation.

Another advantage of mNGS is that it is comprehensive and low-biased. The range of mNGS results covers 6350 species of bacteria, 1798 DNA viruses, 1064 fungi, and 234 species with known genome sequences. Bacteria, 1798 DNA viruses, 1064 fungi, and 234 parasites. Parasites, essentially including all spinal infection pathogenic microorganisms. Although the difficulty of DNA extraction from different species, the bias of the test compared with the conventional test. However, the bias is low compared with conventional tests. Theoretically, it is possible to directly obtain all the genetic information in the specimen. It excludes the subjective purpose and intervention of the detector, which makes up for the tendency and limitation of various single-laboratory tests. And limitations of various single-laboratory tests. At the same time, different from alveolar lavage fluid, pus, and other specimens of infectious diseases, the presence of unclear foci and localized contaminants affect the interpretation of mNGS results, the spine is a naturally sterile environment. The spine is a natural sterile environment, and the foci are relatively clear and confined after the occurrence of infection. Therefore, when mNGS detects rare and uncommon pathogenic microorganisms, it usually has higher accuracy and confidence. In this study, mNGS detection identified multiple infections, anaerobic infections, specific types of infections, and other rare microbial infections were identified by mNGS, and the diagnosis was confirmed clinically by combining history, imaging, special laboratory tests, and diagnostic treatment.

Compared with spinal puncture biopsy, spinal endoscopic lesion removal and sampling can provide enough tissue for smear tests, microbial culture using various culture media, and pathological examination. This ensures a comprehensive clinical testing program, reducing the risk of missed diagnosis and misdiagnosis. While taking samples, we also removed the lesion. During the operation, we cleared out pus, pathogenic bacteria, inflammatory factors, and necrotic tissues by using large amounts of saline for rinsing. As a result, the patient’s postoperative back and leg pain was immediately relieved.

## Conclusion

Spinal endoscopic debridement combined with metagenomic next-generation sequencing (mNGS) for the diagnosis and treatment of primary spinal infections combines the advantages of endoscopic surgery for direct lesion removal and mNGS for precise and rapid diagnosis, which can achieve favorable clinical outcomes. However, this study still has the following shortcomings: (1) This study was a single-center retrospective study, and the evidence level was lower than that of multi-center, prospective controlled studies; (2) Compared with open specimens, the access range of minimally invasive specimens is limited, which may affect the detection rate of pathogens; (3) This study only preliminarily compared the use of antibiotics before collection of minimally invasive specimens and open specimens, and did not conduct accurate statistical analysis on the time of drug withdrawal before surgery, which may cause certain bias in the results.

## Data Availability

No datasets were generated or analysed during the current study.
